# Finite element simulation and clinical follow-up of lumbar spine biomechanics with dynamic fixations

**DOI:** 10.1371/journal.pone.0188328

**Published:** 2017-11-29

**Authors:** Yolanda Más, Luis Gracia, Elena Ibarz, Sergio Gabarre, Diego Peña, Antonio Herrera

**Affiliations:** 1 Department of Mechanical Engineering, University of Zaragoza, Zaragoza, Spain; 2 Aragón Institute of Engineering Research, Zaragoza, Spain; 3 Spine Unit, Department of Orthopaedic Surgery and Traumatology, Miguel Servet University Hospital, Zaragoza, Spain; 4 Aragón Health Research Institute, Zaragoza, Spain; 5 Department of Surgery, School of Medicine, University of Zaragoza, Zaragoza, Spain; University of Crete, GREECE

## Abstract

Arthrodesis is a recommended treatment in advanced stages of degenerative disc disease. Despite dynamic fixations were designed to prevent abnormal motions with better physiological load transmission, improving lumbar pain and reducing stress on adjacent segments, contradictory results have been obtained. This study was designed to compare differences in the biomechanical behaviour between the healthy lumbar spine and the spine with DYNESYS and DIAM fixation, respectively, at L4-L5 level. Behaviour under flexion, extension, lateral bending and axial rotation are compared using healthy lumbar spine as reference. Three 3D finite element models of lumbar spine (healthy, DYNESYS and DIAM implemented, respectively) were developed, together a clinical follow-up of 58 patients operated on for degenerative disc disease. DYNESYS produced higher variations of motion with a maximum value for lateral bending, decreasing intradiscal pressure and facet joint forces at instrumented level, whereas screw insertion zones concentrated stress. DIAM increased movement during flexion, decreased it in another three movements, and produced stress concentration at the apophyses at instrumented level. Dynamic systems, used as single systems without vertebral fusion, could be a good alternative to degenerative disc disease for grade II and grade III of Pfirrmann.

## Introduction

A lot of patients suffer low back pain in some of them with chronic evolution. Lumbar pain can have multiple etiologies, in some cases unidentified. One of the most prevalent etiologies of lumbar pain is degenerative disc disease (DDD) [1]. The etiology of DDD is multifactorial, in its production they influence, among other: the age, sedentary lifestyle, toxic habits, obesity [2], loads supported [3] which in addition can activate the inflammatory and enzymatic processes which play an important role in the degeneration [4–6] movements during flexion [7] and the genetics of each individual [8, 9], with particular relevance of the genetic polymorphisms [10, 11].

Most patients exhibit grades IV and V of Pfirrmann [12] in magnetic resonance imaging (MRI) and evident signs of facet arthrosis leaving instability as the only remaining aspect to improve with surgical treatment.

Lumbar spinal fusion is a standardized and widely accepted procedure for the treatment of discogenic back pain, showing good results in the long term. It can be achieved through anterior lumbar interbody fusion (ALIF), postero-lumbar interbody fusion (PLIF), transforaminal lumbar interbody fusion (TLIF), lateral lumbar interbody fusion (XLIF), non-instrumented posterolateral fusion and circumferential interbody fusion by double approach (anterior and posterior).

The question which remains is if adjacent segment disease (ASD) is produced by age-related degeneration or if it is a consequence of the previous fusion. [13]. As it has been reported, ASD has a multifactorial etiology [13–17]. In order to avoid or minimize the occurrence of ASD, several alternative techniques in the treatment of disc degeneration (DD) have emerged including: arthroplasty of facet joints [18], total disc replacement (TDR) and dynamic fixation (DF). Nowadays, DF is the most used among these techniques.

Dynamic fixations can be used as a surgical treatment system for degenerative disc disease or as a hybrid system, combined with circumferential fusion, to reduce a further progression of degeneration in the adjacent discs to fusion [19–21]. During last years, the two most employed systems are: the DIAM fixator (Device for Intervertebral Assisted Motion) [22], used as an interspinous spacer and the dynamic neutralization system (DYNESYS) [21], although nowadays Dynesys continues being used and the use of interspinous spacers has diminished, including DIAM.

Although in vitro and clinical studies in the mid-term reported good results using DIAM [23, 24] together with a lower incidence of ASD [25], it presented a high rate of revision surgery due to either the loosening or fracture of the interspinous apophyses [26]. Regarding the DYNESYS system, some follow-up studies have reported evident clinical improvement in patients [21, 27].

The biomechanical analysis of the wide variety of fixations can be developed through in vitro and in vivo testing and through finite element (FE) simulations. Concerning in vitro tests, some authors have studied rigid and semi-rigid implants: Wallis, DYNESYS, Locker implant and pedicle screw rod [28, 29], in terms of range of motion (ROM) and at different levels of fixation [30]. Regarding in vivo studies, clinical outcome was evaluated for DIAM dynamic stabilization with successful results in ROM and intervertebral fusion [31]. Several FE studies have been developed to study the effect of implant positioning [32], different flexible implants (FlexPLUS, DSS, DYNESYS, NFlex and PEEK, Awesome Rod System) and rigid fixations evaluating ROM in the four principal movements [33–37], the effect of pre compression level, tension at the screws [38, 39] and several other parameters: Young’s modulus, diameter of the cords, angular stiffness of head screws, etc [40]. The influence of the spacer diameter of DYNESYS on lumbar spine biomechanics was analyzed in a previous study [41].

Despite the number of studies published, contradictory results have been obtained for the different fixations. Thus, the aim of this work is to analyse and compare differences in the biomechanical behaviour between the healthy lumbar spine model and the two operated models with dynamic fixations at L4-L5 level, evaluating the efficiency of dynamic implants in terms of mobility recovery against the healthy spine, used as baseline, and verifying the influence on biomechanical behaviour of adjacent segments. With respect to previous works, the main contribution is the combination of FE study and clinical results in our patients operated with both systems, covering a total of 58 operated patients, with important low back pain, without clinical improvement after several months of conservative treatment, and with Pfirrmann II and III of disc degeneration [12], without instability or signs of vertebral arthrosis. The clinical study allowed verifying if the use of dynamic fixations is able to avoid or diminish the appearance of the ASD, in the non-operated intervertebral discs, which is one of the main reasons for the use of DF, completing and confirming, if possible, the results obtained by means of FE simulations. The present study refers to the use of dynamic fixation as single systems without vertebral fusion (not hybrid systems).

## Material and methods

### Computational simulations

The present work is based on a previous FE model [42] validated according to the four basic movements for a healthy spine (flexion, extension, lateral bending and axial rotation). This model was used as a baseline to generate two new models of lumbar spine with arthrodesis using two types of dynamic fixation and thus compare results from simulations of healthy and implanted models (DYNESYS and DIAM, at L4-L5).

**Fig 1:**
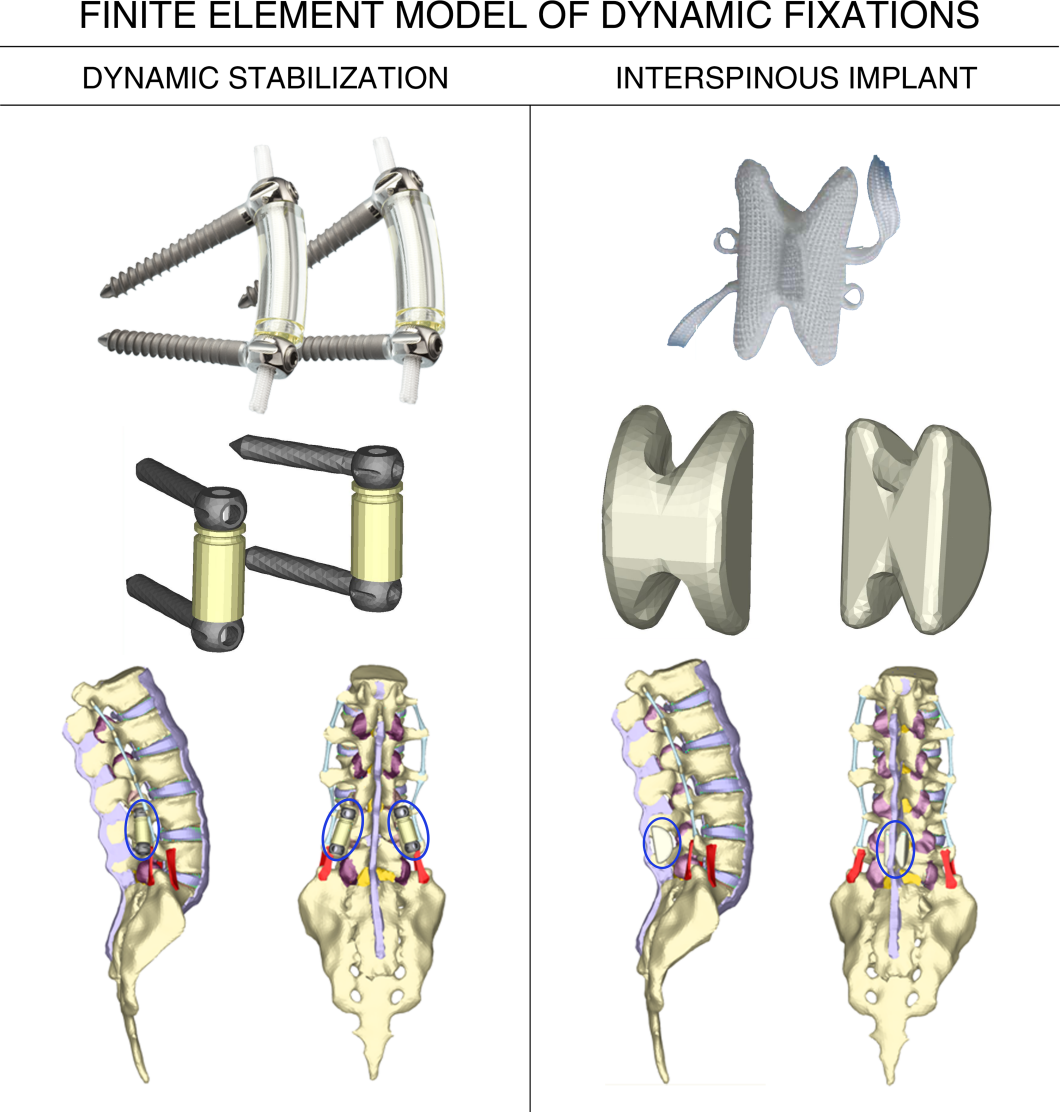
Analyzed devices, their FE models and lateral and posterior view of the operated models. (A) DYNESYS. (B) DIAM.

The DYNESYS system (Zimmer GmbH Warsaw, Indiana, USA) (Fig 1) has two cylindrical space bars made of Polycarbonate-urethane (Selene PCU) which fasten spinal segments towards anatomical position absorbing loads and controlling the spine through extension positions; a stabilizer cord made of Polyethylene-terephthalate (Selene PET) which under traction, compresses the space bar achieving the global stability of the whole implant; and for pedicle locking screws made of a Titanium-Aluminium-Niobium alloy (Ti-Al-Nb) (Protasul-100), which permit the fixation of the cord and enable the compression of the modular space bars.

The DIAM Spinal Stabilization System (Medtronic, Minneapolis, USA) (Fig 1) consists of a silicone structure covered by a polyester H-shape mesh placed in between the spinous apophyses, acting as a shock absorber reducing loads at the vertebrae and serving as a flexible support for the lumbar spine through its degenerative process. It is fixed in position by two cords serving as bonds. The DIAM device was conceived to stabilize degenerations of spine segments without fusion surgery, maintaining the movement of the segments and preventing the degeneration of adjacent segments.

Three models, one for the healthy column and two with fixations were generated and meshed. Implants were meshed automatically with linear tetrahedra except for the cords and the space bars which were meshed with linear hexahedra. Thereafter, the positioning of each device on the healthy lumbar spine model was carried out. Concerning the DYNESYS model, screws were inserted in the perforations prepared during the initial stage, provided that interdiscal space had not been affected by disc degeneration. Respecting the DIAM model, device insertion was executed by posterior approach removing supraspinous ligament between L4 and L5 vertebrae to locate the DIAM device between the spinous apophyses. The final models with arthrodesis are shown in Fig 1.

The 3D FE model of the healthy lumbar spine consisted of 195726 elements. The statistics of the implanted models are shown in Tables 1 and 2. The final mesh sizes for the three models were obtained after performing a sensitivity analysis, refining the mesh in order to achieve a convergence towards a minimum of the potential energy, both for the whole model and for each of its components, with a tolerance of 1% between consecutive meshes.

**Table 1:** Number and type of elements of each component in the FE models with DYNESYS and DIAM, respectively.

Group	Element type	Number of elements Dynsesys model L4-L5	Number of elements DIAM model L4-L5
Cartilage	Wedge	4077	3086
Anterior longitudinal ligament	Wedge	9967	9046
Posterior longitudinal ligament	Wedge	4115	3844
Ligamentum flavum	Tetrahedron	2619	3042
Intertransverse ligament	Tetrahedron	7016	6678
Capsular ligament	Membrane	2039	3225
Interspinous ligament	Tetrahedron	2972	2363
Supraspinous ligament	Tetrahedron	2770	2611
Iliolumbar ligament	Wedge	822	816
Annulus fibrosus	Hexahedron	8288	8288
Nucleus pulposus	Tetrahedron	14410	14410
Annulus fiber layers 1	Truss	592	592
Annulus fiber layers 2	Truss	592	592
Annulus fiber layers 3	Truss	592	592
Annulus fiber layers 4	Truss	592	592
Annulus fiber layers 5	Truss	296	296
Outer vertebral endplates	Tetrahedron	6507	3578
Intermediate vertebral endplates	Tetrahedron	4047	2244
Center of the vertebral endplates	Tetrahedron	2055	831
Walls of the vertebral body	Tetrahedron	52456	37205
Cancellous bone (inside vertebrae)	Tetrahedron	64038	44133
Posterior vertebra	Tetrahedron	51416	47134
Spinal stabilization system	See Table 2	16402	11099
**Total**		**251811**	**258680**

**Table 2:** Material properties of every fixation component.

Device	Component	Material	Young Modulus (MPa)	Poisson’s ratio	N° of elements/Element type
DYNESYS (L4-L5)	Screws	Protasul 100 (Ti-Al-Nb alloy)	110000	0.33	11375/Tetrahedron
	Space bars	Poliethylene-terefthalate	1980 (*)	0.35	2420/Hexahedron
	Cord	Poliethylene-terefthalate	3225 (*)	0.40	2607/ Hexahedron
Interspinous fixation	DIAM	Silicone core covered by polyester	2100	0.35	11099/Tetrahedron

(*) Obtained from experimental testing

Regarding the model with DYNESYS, once pedicular screws were fixed, the length of the space bar was determined and the cord was inserted applying a pre-compression to simulate stretching with a value of 50 N (usual value in surgical practice). To this respect, the options Rebar and Initial Conditions in Abaqus [43] were used, allowing changing the pre-stress state during the subsequent equilibrating static analysis steps. Polyester laces placed at each side of the DIAM device were not modelled, as their restraint effect was included as a boundary condition.

**Fig 2:**
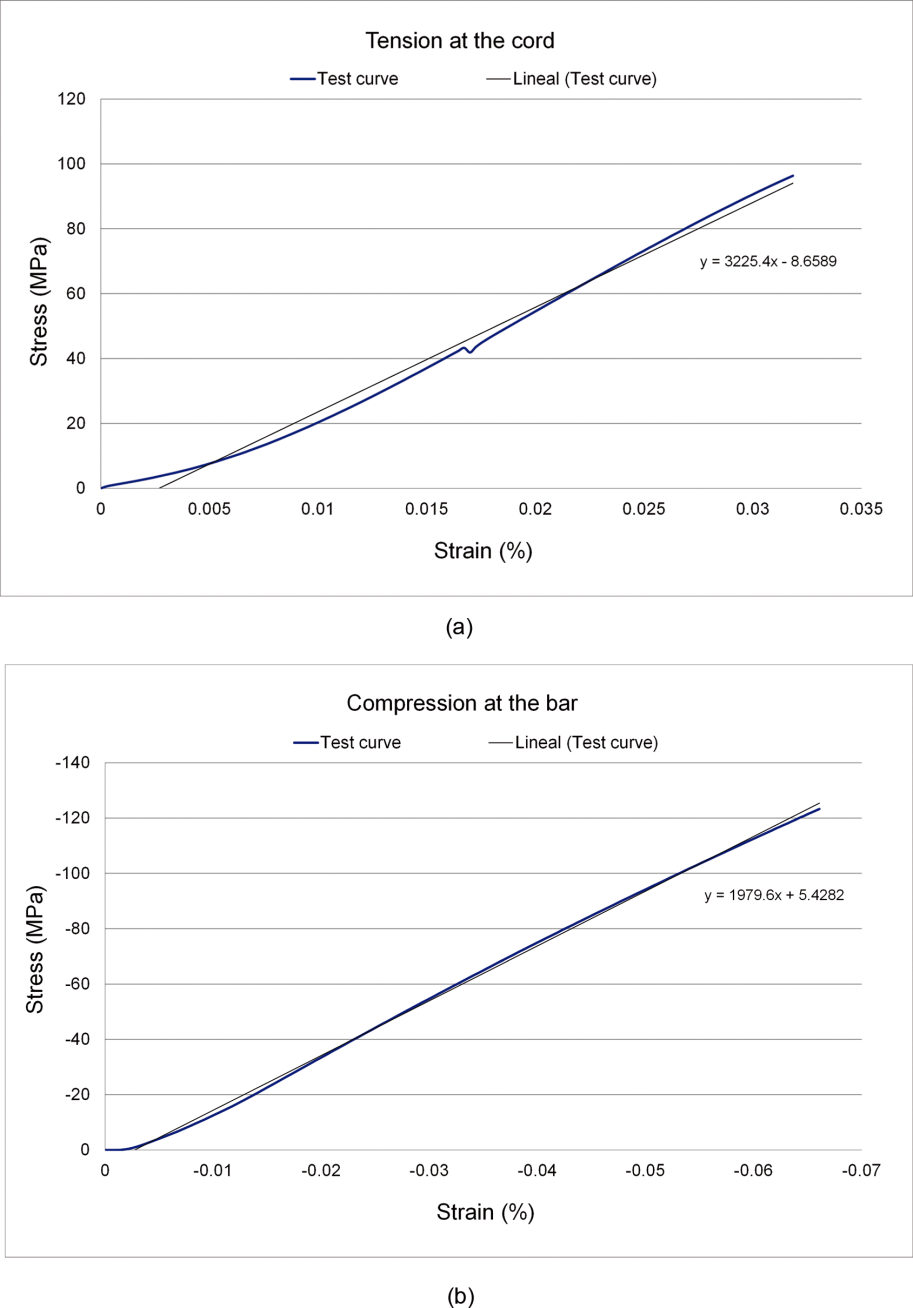
Experimental strain-stress curves for DYNESYS device. (A) Cord. (B) Bar.

For the DYNESYS model, tie constraints [43] were applied between the screws and the bone and the screws and the cords (i.e., perfectly bonded). Conversely, contact interaction was considered between the space bar and the cords (friction coefficient of 0.1) and the space bar and the screws (friction coefficient of 0.1). The DIAM model included contact interaction between the polyester mesh and the bone (friction coefficient of 0.2). Mechanical properties of biological tissues corresponded to those included in a previously published study [42]. DYNESYS components were made from the following materials: screws were made of Protasul 100 Ti-Al-Nb alloy, space bars were made of Poliethylene-terephthalate, and the cord of Poliethylene-terephthalate. Mechanical properties of the cord and space bar of the DYNESYS device were obtained by traction and compression tests, respectively, using an Instron Axial-Torsion Servohydraulic Fatigue Testing System (model 8874) at the Mechanical Engineering Lab of the University of Zaragoza. To prevent sliding, a set of load-displacement curves were obtained. A quasi-static traction test was performed with a displacement rate of 2 mm/min and an extension limit of 2 mm. The space bar was tested under a compression rate of 1 mm/min and 3 mm of compression limit. The stress and strain values obtained for the cord and space bar in the traction test and compression test are shown in Fig 2A and 2B, respectively. Regarding Interspinous fixation DIAM, it was made of Silicone core covered by polyester.

For the affected disc, in the models with DYNESYS and DIAM, mechanical properties according to Ibarz [44] were considered (equivalent to grade II or III of Pfirrmann [12], which corresponds to slightly affected vertebral endplates, in grade III, but without instability. This is in accordance with clinical recommendations for dynamic fixations.

The boundary conditions applied were the same in every model: movements at the sacrum wings were restrained, and a pure moment scenario as reported in [45] was considered. A unified moment of 7.5 Nm was applied at the central node on the top side of the vertebra L1 around the corresponding axes for flexion, extension and lateral bending, and a moment of 0.6 Nm for axial rotation. Those values are in accordance with the recommended ones for the lumbar spine in pathological conditions [40].

**Fig 3:**
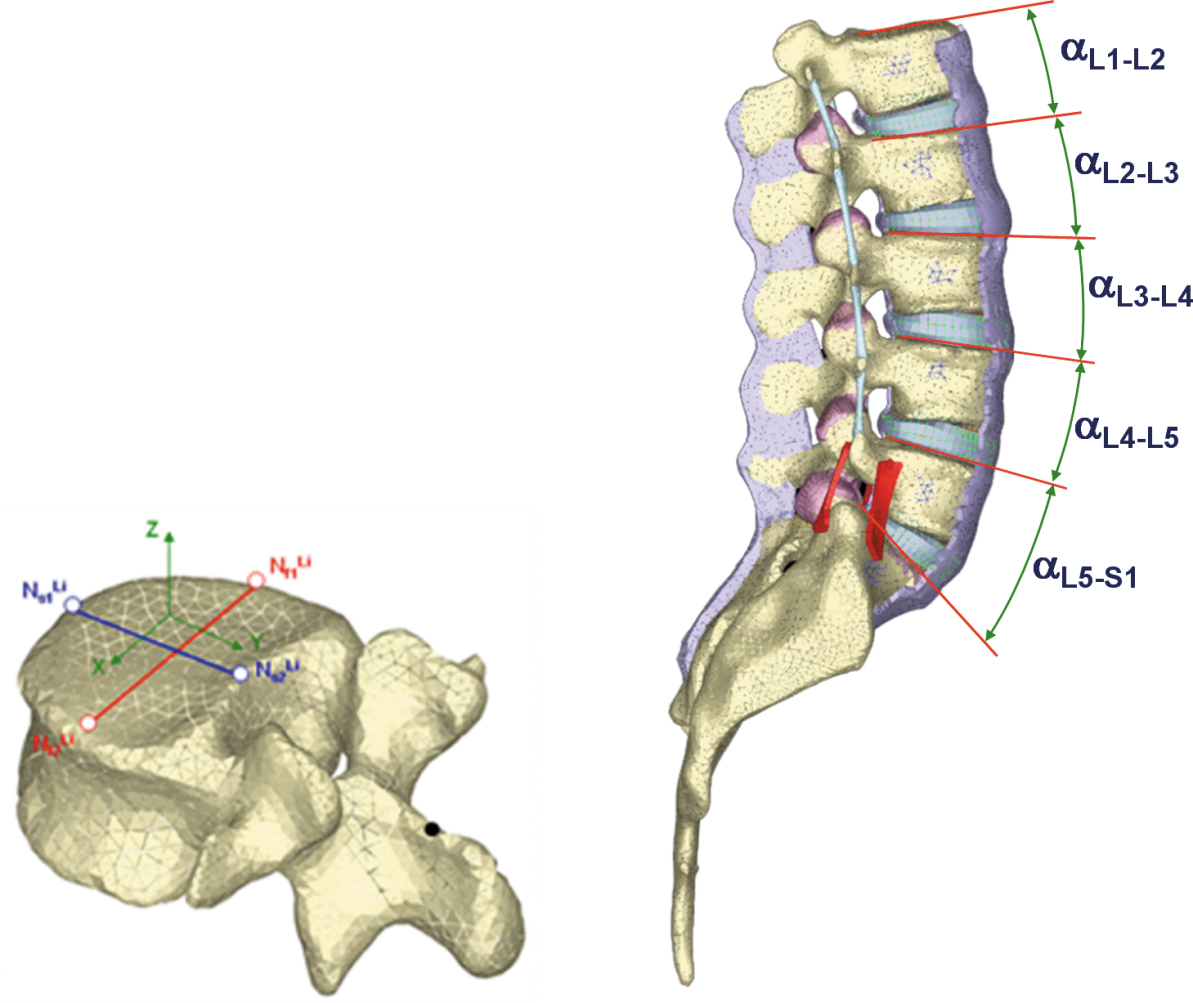
Geometrical references for relative movement calculation.

In order to analyse the angles formed by each vertebra with the sacrum in the four basic movements, the same technique described in [42] was used (Fig 3). Flexion and extension movements were analysed by measuring the angle between facet joints at the sagittal plane; lateral bending movement was analysed by measuring the angle between facet joints at the frontal plane. Finally, axial rotation movement was analysed by measuring the angle between facet joints at the coronal plane.

### Clinical study

A total of 58 patients were operated on for degenerative disc disease, 46 patients with a DIAM device and 12 patients with DYNESYS, all of whom were examined to know their evolution and the long-term results of surgical treatment. This study was authorized by the Ethics Committee of Aragon and all patients signed the corresponding informed consent. Both groups of patients were similar, in age and degree of disc degeneration.The inclusion criteria were:

- Patients younger than 50 years
- Persistent chronic low back pain after at least 6 months of conservative treatment
- Pfirrmann Grade II or III of degenerative disc disease
- No existence of facet osteoarthritis or instability
- No previous surgery

The exclusion criteria were:

- Patients older than 50 years
- Pfirrmann Grade IV or V of Degenerative Disc Disease
- Existence of facet osteoarthritis or instability
- Previous surgeries

The study of patients operated with Dynesys was retrospective, the assessment of outcome was performed by an independent observer. The study of patients operated with DIAM was prospective.

## Results

### Results of computational simulations

**Fig 4:**
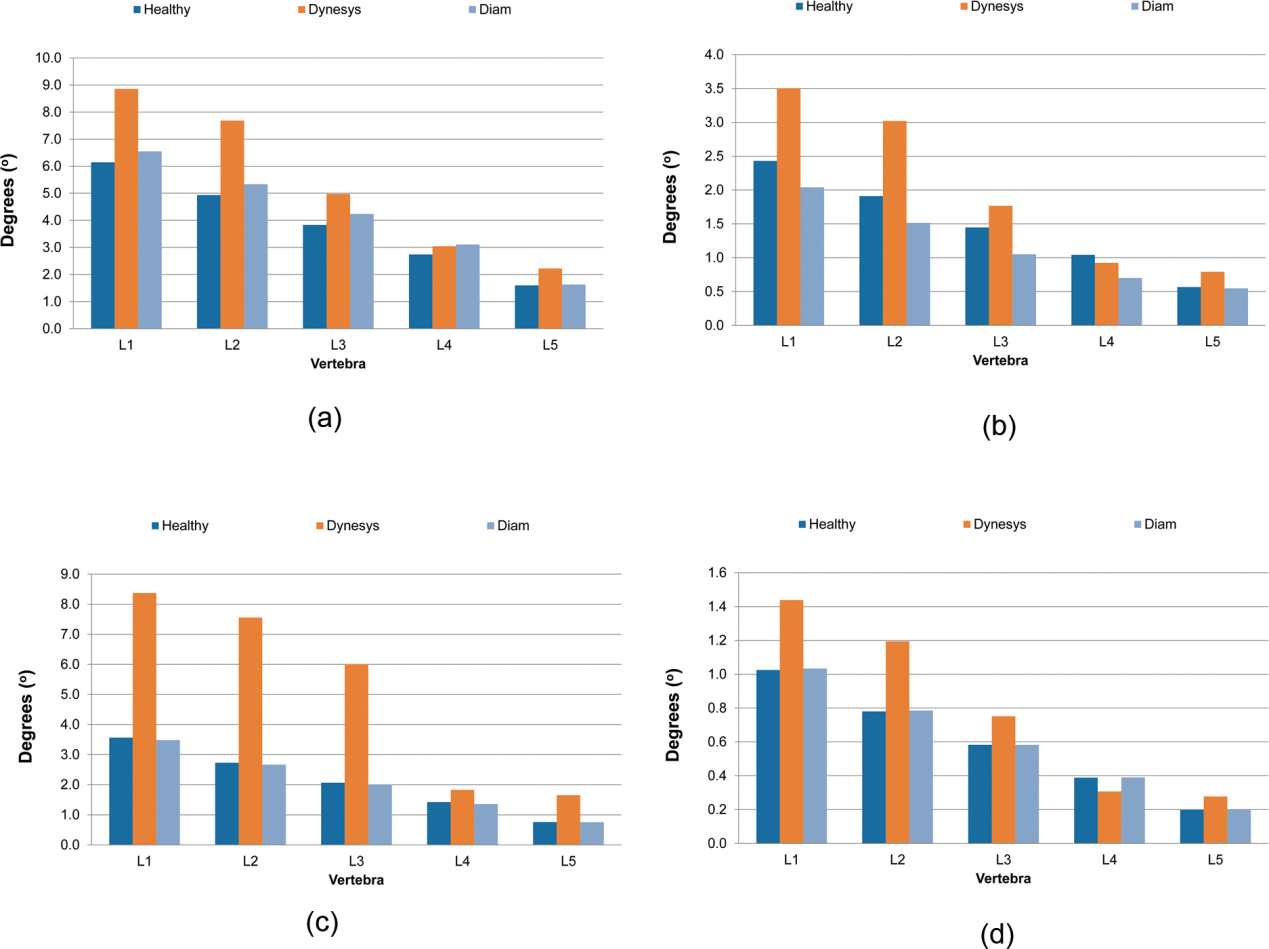
Results of the movement angle at each vertebra. (A) Flexion. (B) Extension. (C) Lateral bending. (D) Axial rotation.

Concerning the results of FE simulations, the mobility values shown in Fig 4 were obtained. Regarding flexion, Fig 4A shows similar behaviour between the healthy and interspinous device model along all the vertebrae. The DYNESYS device model was not as stiff as the healthy one yielding to a greater global movement.

In extension movement (Fig 4B), the DYNESYS device provided a higher movement as pre compression of the cord favoured this type movement whereas for the interspinous model a higher mobility was developed at superior segments (L1 and L2).

Regarding lateral bending movement in undeformed and deformed shapes, the DYNESYS model had a marked higher movement compared to the other models. The interspinous model gave a similar ROM to the healthy one (Fig 4C).

Concerning rotation axial movement (Fig 4D), lower amplitude was observed compared to previous movements. The healthy and DIAM model had a similar ROM again with the range being more reduced for the DYNESYS model.

Table 3 summarises all the results concerning the implanted models compared with the healthy model. The DIAM device produced, in general, smaller variations in degree of movement compared to DYNESYS, exhibiting minimal variations for axial rotation (+0.10/+0.78%) and lateral bending (-0.48/-4.59%). In extension it increased +13.35% with respect to the healthy model reaching a maximum variation for flexion (-32.65%) where all variations were negative as in lateral bending. Consequently, movement compared to the healthy model was limited. Conversely, DIAM increased movement along all the vertebrae during extension. Vertebra L5 (1.86%) had the smallest variation, corresponding to the movement of extension, whereas L4 (-32.65%) was the most altered one, in the movement of flexion.

**Table 3:** Variations of the degree of movement compared to healthy model through the four movements simulated (% of relative rotation between vertebrae).

Movement	Implant	L1	L2	L3	L4	L5
Flexion	DYNESYS	44.13	58.07	22.10	-11.36	40.03
	DIAM	-16.12	-20.76	-27.33	-32.65	-3.21
Extension	DYNESYS	44.05	55.82	29.85	10.94	39.05
	DIAM	6.50	8.15	10.53	13.35	1.86
Lateral bending	DYNESYS	135.07	176.84	190.86	28.47	117.60
	DIAM	-2.22	-2.28	-2.93	-4.59	-0.48
Rotation	DYNESYS	40.20	53.20	28.98	-21.07	39.79
	DIAM	0.78	0.63	0.10	0.33	-0.35

Compared to the healthy model, the DYNESYS device produced higher variations in the degree of motion, reaching a maximum value during lateral bending. Flexion and extension produced the same range of variations. The same range of percentages is reached for rotation movement. In summary, vertebra L4 (10.94%) was the least altered, occurring in the movement of extension, whereas L3 (190.86%) experimented the highest variation, corresponding to lateral bending.

**Fig 5:**
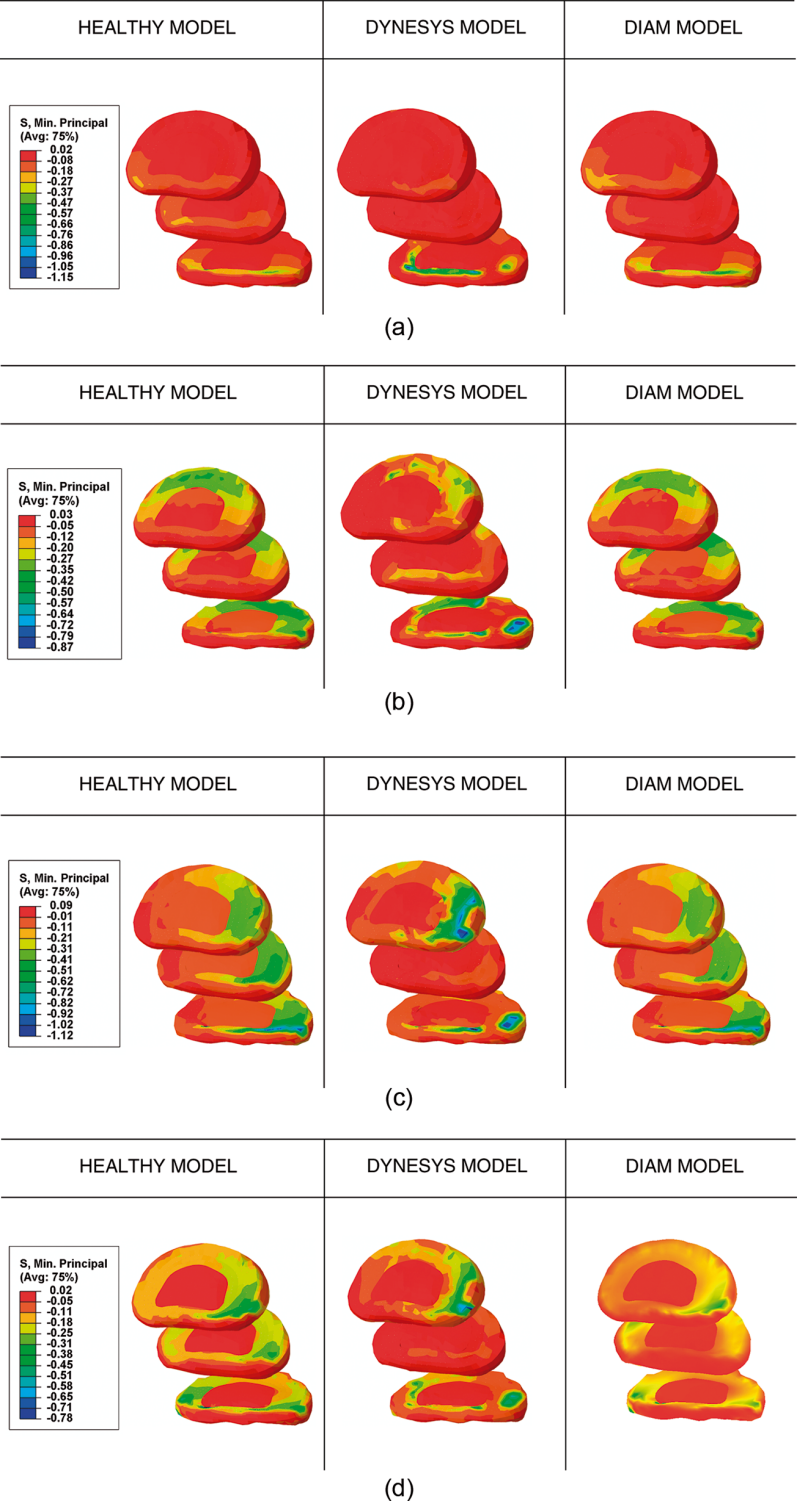
Compression stress maps in discs L3- L4 and L5-S1 for healthy, DYNESYS and DIAM models. (A) Flexion. (B) Extension. (C) Lateral bending. (D) Axial rotation.

Interdiscal pressure (IDP) was measured as compressive stresses (minimum principal stress) at the instrumented and adjacent levels (Fig 5). During flexion, DYNESYS decreased IDP mainly at the instrumented level (L4-L5) and slightly at the upper level (L3-L4) whereas it slightly increased IDP at the lower level (L5-S1). Conversely, with the DIAM device IDP remained stable at every level (Fig 5A). In extension, DYNESYS increased IDP at the lower level (L5-S1) without producing any changes to the rest of discs, while IDP continued to be stable with DIAM at every level (Fig 5B). In lateral bending, IDP decreased at the instrumented level but increased in adjacent discs with DYNESYS, whilst remaining stable with DIAM at every level (Fig 5C). Finally, in axial rotation, IDP decreased at the instrumented and lower level and increased in the upper disc with DYNESYS, whereas a slight discharge was produced at every level with DIAM (Fig 5D).

**Fig 6:**
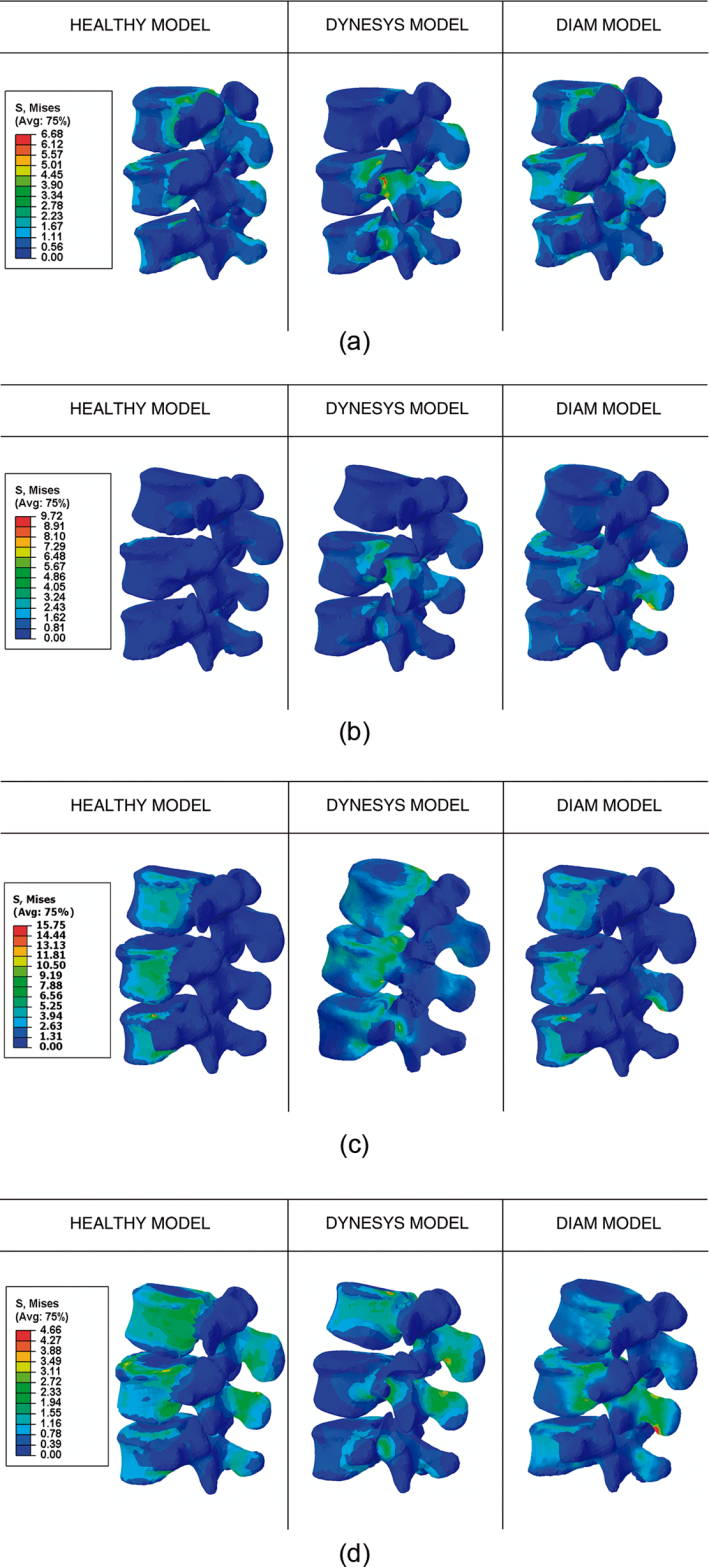
Von Mises stress maps in vertebras L3, L4 and L5 for healthy, DYNESYS and DIAM models. (A) Flexion. (B) Extension. (C) Lateral bending. (D) Axial rotation.

Results concerning the simulated movements are post processed at L3-L4-L5 in terms of von Mises stress, as Fig 6 shows. Depending on the fixation used, maximum values were concentrated at the location of the fixations: the DYNESYS implant at the screw insertions in contrast to the DIAM at the apophyses. The von Mises criterion was used for comparison purposes only, and it cannot be used as failure criterion for bone, which has a brittle behavior.

In flexion movement (Fig 6A) the DYNESYS implant produced higher stress concentrations at the screw insertions at L4-L5 particularly at vertebra L4. L3 was discharged which is related with the onset of screw loosening. The DIAM implant developed a similar behaviour to the healthy model but overloading the apophyses of L4.

Regarding extension movement (Fig 6B), DYNESYS showed a high stress rate at the insertion screws of L4 while L3 remained almost unloaded. The DIAM implant overloaded both L4 and L5, a stress concentration is observed at the apophyses of L4 vertebra.

During lateral bending (Fig 6C), the DIAM device transmitted loads mainly through the apophyses of L4. In relation to DYNESYS, L4 and L5 had a similar stress distribution to the healthy model, except at the screw insertions where a concentration appears again. L3 had a noticeable increase of stress compared to the healthy model, with a different distribution and higher values near to the back vertebral body.

Axial rotation discharged L4 and L5 in the DYNESYS model while this movement overloaded the apophyses of L3 and L4. The DIAM device exhibited a similar distribution to the healthy model, except at the apophyses of L4 which exhibited a stress concentration and at L3 where a slight decrease was observed (Fig 6D).

### Results of clinical study

#### DIAM

Between the years 2012 and 2016, 46 patients were operated on, 3 of them with a double device in L4-L5 and L5-S1. The ages range from 26–43 years with an average of 35.7 years. The follow-up time ranges from 5 years to 8 months with an average of 3.4 years.

Three of them have been reoperated due to persistent low back pain despite conservative treatment. Two at the two-year DIAM placement and another at 14 months, all were treated with lumbar arthrodesis. Of the remaining 43 patients, 6 had lumbar pain and were treated by rhizolysis, and the symptomatology disappeared. The remaining 37 patients (80.43%) were asymptomatic. We have not had any breakage of spinous apophyses nor any displacement of the device. There are no clinical or radiographic signs of ASD in the adjacent segments.

#### DYNESYS

We reviewed 12 patients operated between 2007 and 2010, ranging in age from 28–41 years with an average of 38.3 years. The follow-up time ranges from 10 years to 6.6 years with an average of 8.3 years. Of these 12 patients, 7 are asymptomatic (58.33%), 3 were arthrodesed, 2 of them at 5 years of Dynesys placement and 1 at one year, while the remaining two patients underwent rhizolysis to treat their low back pain, with improvement, only having some occasional discomfort.

We have not detected any pulling of the screws, or breakage of the material. There are no clinical or radiographic signs of ASD in the adjacent segments.

## Discussion

The incidence of clinically symptomatic adjacent segment disease (SASD) is lower compared to radiographic adjacent segment disease (RASD), because radiographic changes in adjacent segments do not necessarily imply functional impairment in patients with Arthrodesis [46].

The incidence of SASD ranging between 2% and 36% [46] and its treatment represents a serious problem, especially in young patients. The stiffness of the implant and the number of fixed segments in lumbar fusion has been associated with an increased incidence of ASD [47]. Nevertheless, fusion produces a significant increase in stress of the adjacent segments, particularly in the facet joints which is considered to provoke a degenerative cumulative process leading to ASD [48].

So as to avoid or minimize the occurrence of ASD, dynamic fixation systems have emerged, becoming a popular alternative to arthrodesis in the treatment of degenerative disc disease. DF reduces IDP at the instrumented levels by unloading the discs [49]. Consequently, an improvement is obtained on the MRI images of the degenerated disc by increasing the proportion of glycosaminoglycans [27].

Dynamic fixations were designed to prevent abnormal motions, yielding to a better physiological load transmission. Posterior motion-sparing devices intend to off-load facet joints and fibrous annulus enabling the damaged discs to repair themselves [50] if the degenerative process is not very advanced. They improve lumbar pain and reduce the stress on adjacent segments. Computational biomechanical research has confirmed that dynamic systems protect adjacent levels from excessive motion [51]. However, other authors consider this technique to produce a high rate (19%) of revision surgeries and low clinical improvement (only 67%) [52].

Our research group has long and mid-term experience with DF (DYNESYS and DIAM) for the treatment of degenerative disc disease localized at L4-L5 level. Important clinical symptoms and clear signs of disc degeneration were confirmed by MRI, but without instability or degeneration of the facet joints in young and active patients. As floating fusion entails a high risk for ASD [53], this technique is considered to be a good alternative.

The four principal movements were simulated. ROM was different in both systems. Both devices allowed motion at L4-L5 level (instrumented level) in flexion without variations in amplitude compared to the healthy spine; in extension, both systems limited mobility, a greater percentage with DIAM; in lateral bending, DYNESYS increased the mobility and DIAM did not produce any variation; finally, in axial rotation, DIAM^TM^ did not alter the range of motion and DYNESYS decreased it by 25%.

Concerning the rest of the segments, the results of the ROM with DYNESYS are in agreement with previous reports [30, 54]; it must be considered that the ROM with DYNESYS may vary in relation to the cord pretension [39], length and diameter of the spacer [41]. The results obtained in the present study with DIAM are in agreement with other works [22].

With respect to the stresses, although neither fixation device produced a significant rise on adjacent vertebrae, they both generated stress concentrations at their locations. Therefore, DYNESYS underwent this increase at the insertion zones of the screws, according to a previous study [54]; the stress concentration can provoke the pull-out of the screws in the medium or long term [21]. The obtained results showed the insertions at vertebra L4 as the most loaded whereas vertebra L3 was discharged, suggesting possible problems related to screw loosening at this level. In our clinical experience, we have not observed any pulling or loosening of the screws.

The DIAM device increased the stresses at the spinous apophyses of the instrumented level. This complication, already described [26], can cause its own fracture. In the 46 operated cases we did not suffer any fracture of the spinous apophyses, although we must consider the follow-up time is not especially long.

The obtained results are in accordance with Wu [26], exhibiting peak stress values at the apophyses of L4 during extension, flexion and lateral bending above all. As a result vertebra L4 remained as the most loaded one. Neither significant increase in mobility in the L5-S1 level nor an increase on the stress were found, considering that a dynamic fixation floating was simulated, like those of our clinical cases.

In the movement of flexion, DIAM and the healthy model developed similar stress maps whereas DYNESYS exhibited the maximum values amongst the three models at the insertion points of L4. During lateral bending the highest range of stresses occurred, where the DIAM model had a very similar stress distribution to the healthy model and the DYNESYS model remained almost discharged. Conversely, the minimum stress values were obtained for axial rotation with the DIAM model exhibiting lower stresses.

Results for IDP are in good agreement with previous published results: DYNESYS decreased IDP at the instrumented level and the facet joint forces at implant level [34] with no significant changes in IDP seen in the adjacent discs [20]. Regarding DIAM, contradictory published results have reported: a decrease in IDP at instrumented level and adjacent discs [48], a decrease in IDP at instrumented level with no significant changes in the IDP at the adjacent levels [55], and only a decrease in IDP during extension at the instrumented level [56]. Nonetheless, a recent paper has published a rise in IDP and the facet load in adjacent segment with an important stress at the bone-implant interface, similar to the findings in the present work [54]. IDP decrease and the stabilization of the stresses on adjacent vertebrae is a positive factor in avoiding the appearance of ASD.

The obtained results did not detect a stress increase on the adjacent segments, which is in accordance with the published evidence where no incidence was observed on ASD after the implantation of dynamic systems. In the same way, our results through FE simulation confirmed a decrease in IDP without any variations in the adjacent discs. In the clinical study we did not observe the presence of ASD in the adjacent discs, which is in favor of that there is no increase of IDP, although in the cases of DIAM the follow-up is short.

Concerning the increased mobility detected in the adjacent segments, particularly with DYNESYS, it does not appear to have clinical implications in the medium term taking into account the published results [21]. The mid-term results obtained by our group in the clinical follow-up were very satisfactory and we did not find hypermobility with displacement of adjacent vertebrae in any case, which is a major cause of ASD [47]. Our clinical results with the use of the DIAM, with 80.43% of asymptomatic patients and without presence of ASD in the adjacent discs, can be considered satisfactory, although more follow-up time is needed to reach definitive conclusions. The results with DYNESYS, with a longer follow-up time and smaller number of patients, are lower, with only 58.33% of asymptomatic patients. But, what is evident is that with both devices we have not detected ASD in the adjacent discs, which is one of the reasons for using dynamic fixations.

The obtained results show that the use of dynamic fixations as single systems without vertebral fusion, for low grades of disc degeneration (grades II and III of Pfirrmann), which corresponds to slightly affected vertebral endplates but without instability, is an advisable technique which can provide good results. Regardin the controversial published results, the results obtained in the present work are in accordance with the authors who consider both systems capable of maintaining the stability of the lumbar spine (DYNESYS [20, 54, 57–60] and DIAM [50, 61–63]).

Our clinical experimental results with DIAM are satisfactory whereas they were not as favorable with the use of DYNESYS, although the latter is considered to provide a more stable fixation. Nonetheless, the follow up period is not long enough to establish definitive conclusions. Both fixations have been used on young patients, and have made it possible to postpone the lumbar spine arthrodesis whenever necessary, as the definitive solution. Additionally, patients have been re-operated after several months of ineffective conservative treatments, recommending a dynamic fixation mainly because of their age.

The main limitations of this study derive from the small sample of patients and the short follow-up time, both of which need to be broadened. Nevertheless, there is a good correspondence between computational results and the absence of ASD in the radiologic controls of the patients.

## Conclusions

The results obtained in the present work are in accordance with other authors who consider both systems (DYNESYS and DIAM) capable of maintaining the stability of the lumbar spine. Nevertheless, it must be noted that the DYNESYS system may have greater long-term stability, whilst also considering that its implementation requires a more aggressive surgery.

Accorfing to the obtained results, the dynamic systems anlyzed, used as single systems without vertebral fusion, could be an alternative for the treatment of degenerative disc disease for grade II and grade III of Pfirrmann. Their major advantage is the possibility they offer to execute a subsequent rigid fixation in case of the failure of the dynamic fixation. In any case, they make it possible to postpone the procedure of lumbar spine arthrodesis.

## Ethical approval

The study “Tratamiento quirúrgico de la discopatía degenerativa de columna lumbar” has been approved by the Ethics Committee of The Institute of Health Sciences of Aragón (protocol number C.P. IACS 43/005-C.I. PI 07/65).

## Supporting information

S1 Fig Analyzed devices, their FE models and lateral and posterior view the operated models. (A) DYNESYS. (B) DIAM.
(ZIP)

S2 Fig Experimental strain-stress curves for DYNESYS device. (A) Cord. (B) Bar.
(TIF)

S3 Fig Geometrical references for relative movement calculation. (TIF)

S4 Fig **A, B, C and D. Results of the movement angle at each vertebra**. (A) Flexion. (B) Extension. (C) Lateral bending. (D) Axial rotation.
(TIF)

S5 Fig **A, B, C and D. Compression stress maps in discs L3- L4 and L5-S1 for healthy, DYNESYS and DIAM models** (A) Flexion. (B) Extension. (C) Lateral bending. (D) Axial rotation.
(TIF)

S6 Fig **A, B, C and D. Von Mises stress maps in vertebras L3, L4 and L5 for healthy, DYNESYS and DIAM models.** (A) Flexion. (B) Extension. (C) Lateral bending. (D) Axial rotation.
(TIF)

S1 Table Number and type of elements of each component in the FE models with DYNESYS and DIAM, respectively. (TIF)

S2 Table Material properties of every fixation component. (TIF)

S3 Table Variations of the degree of movement compared to healthy model through the four movements simulated (% of relative rotation between vertebrae). (TIF)

## Abbreviations

3D: Three-Dimensional
DDD: Degenerative disc disease
MRI: Magnetic Resonance Imaging
ALIF: Anterior lumbar interbody fusion
PLIF: Postero-lumbar interbody fusion
TLIF: Transforaminal lumbar interbody fusion
XLIF: lateral lumbar interbody fusion
ASD: Adjacent Segment Disease
DD: Disc Degeneration
TRD: Total disc replacement
DF: Dynamic fixation
DIAM: Device for intervertebral assisted motion
ROM: Range of motion
FE: Finite element
PEEK: Polyether ether ketone
PCU: Polycarbonate-urethane
PET: Polyethylene-terephthalate
IDP: Interdiscal Pressure
L1: First lumbar vertebrae
L2: Second lumbar vertebrae
L3: Third lumbar vertebrae
L4: Fourth lumbar vertebrae
L5: Fifth lumbar vertebrae
S1: First sacral vertebrae
SASD: Symptomatic Adjacent Segment Disease
RASD: Radiographic Adjacent Segment Disease
