# Supplementary figures and images for: Finite element simulation and clinical follow-up of lumbar spine biomechanics with dynamic fixations
Source: PLoS One. 2017 Nov 29;12(11):e0188328. doi: 10.1371/journal.pone.0188328 (PMC5706716; doi:10.1371/journal.pone.0188328)

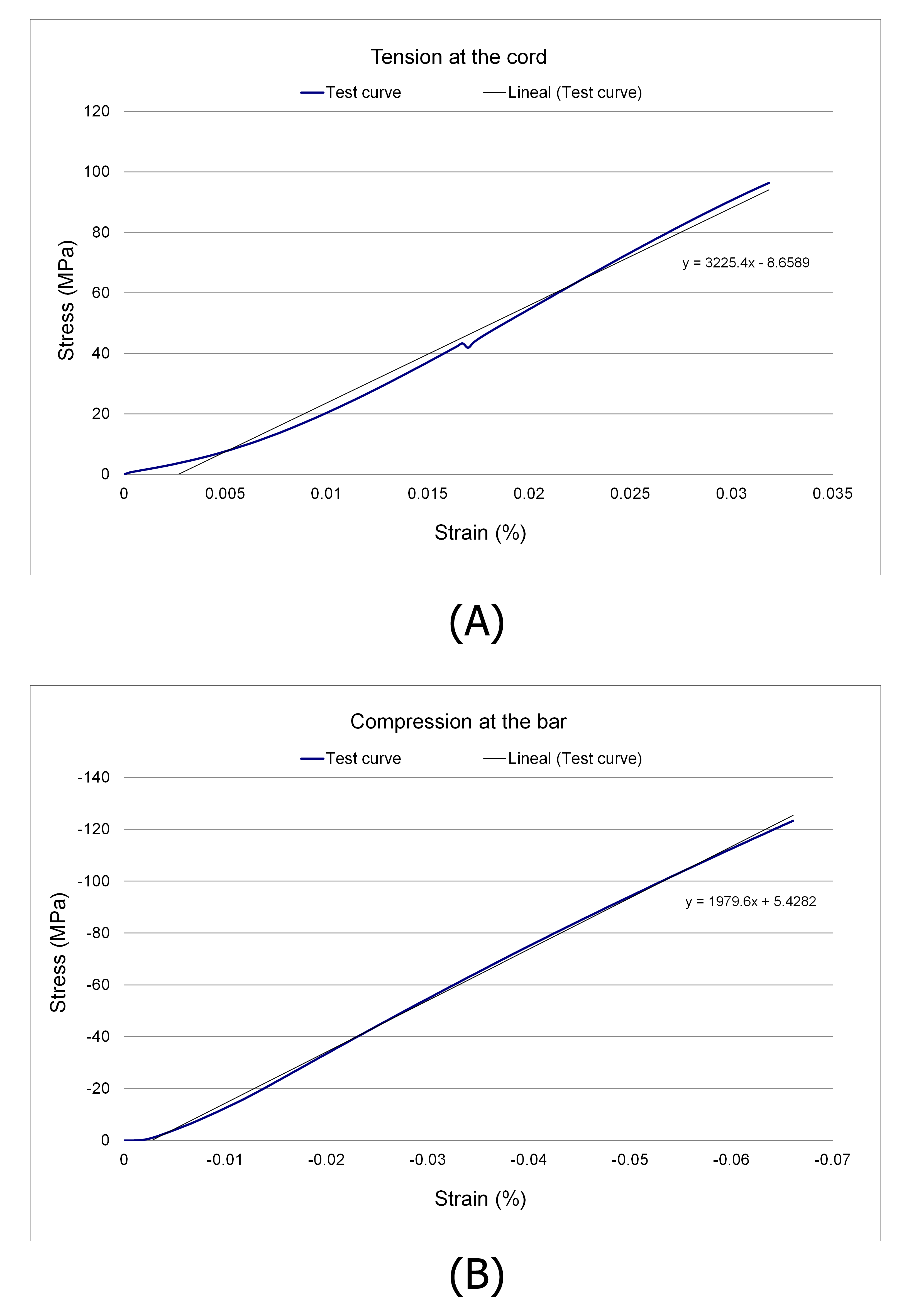

Supplement: S2 Fig — (A) Cord. (B) Bar. (TIF) [file pone.0188328.s002.tif]

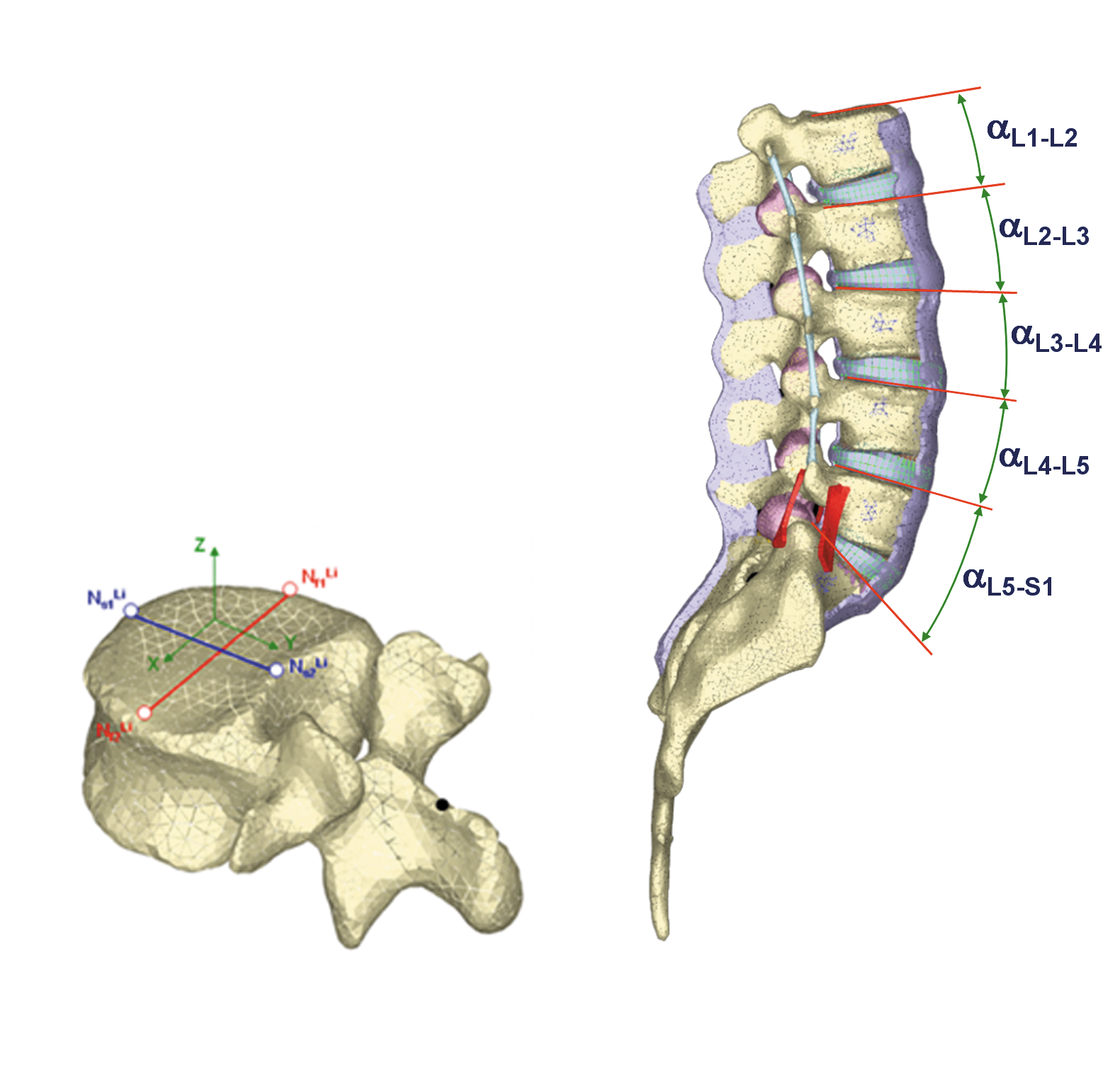

Supplement: S3 Fig — (TIF) [file pone.0188328.s003.tif]

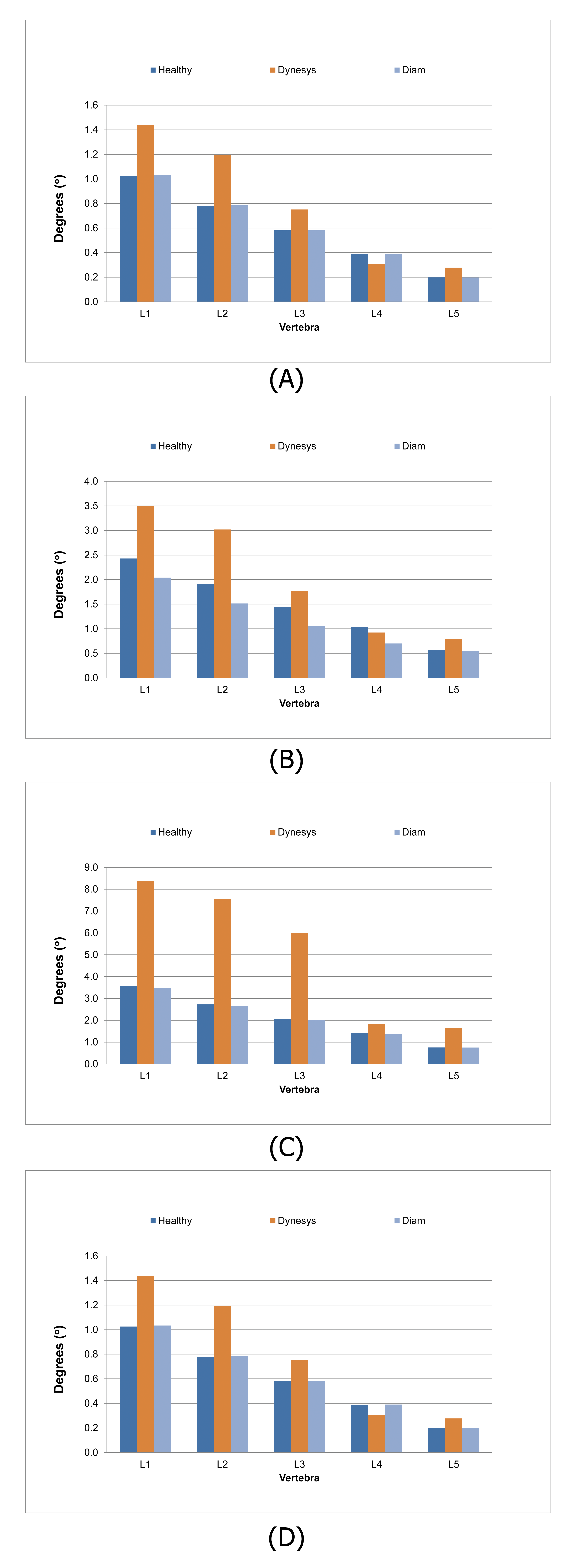

Supplement: S4 Fig — A, B, C and D. Results of the movement angle at each vertebra. (A) Flexion. (B) Extension. (C) Lateral bending. (D) Axial rotation. (TIF) [file pone.0188328.s004.tif]

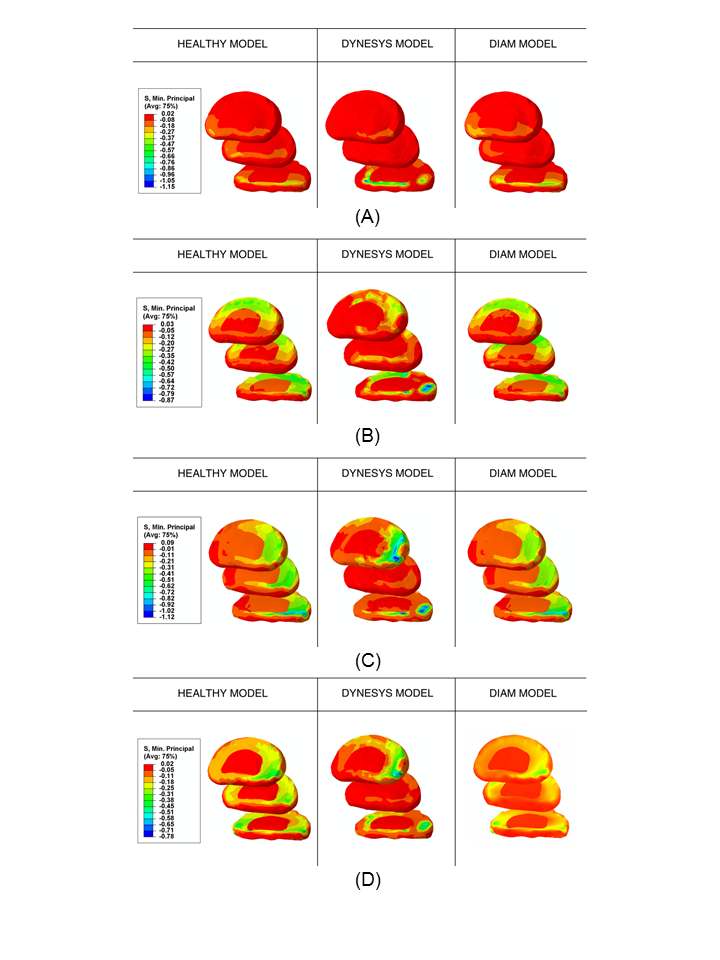

Supplement: S5 Fig — A, B, C and D. Compression stress maps in discs L3- L4 and L5-S1 for healthy, DYNESYS and DIAM models (A) Flexion. (B) Extension. (C) Lateral bending. (D) Axial rotation. (TIF) [file pone.0188328.s005.tif]

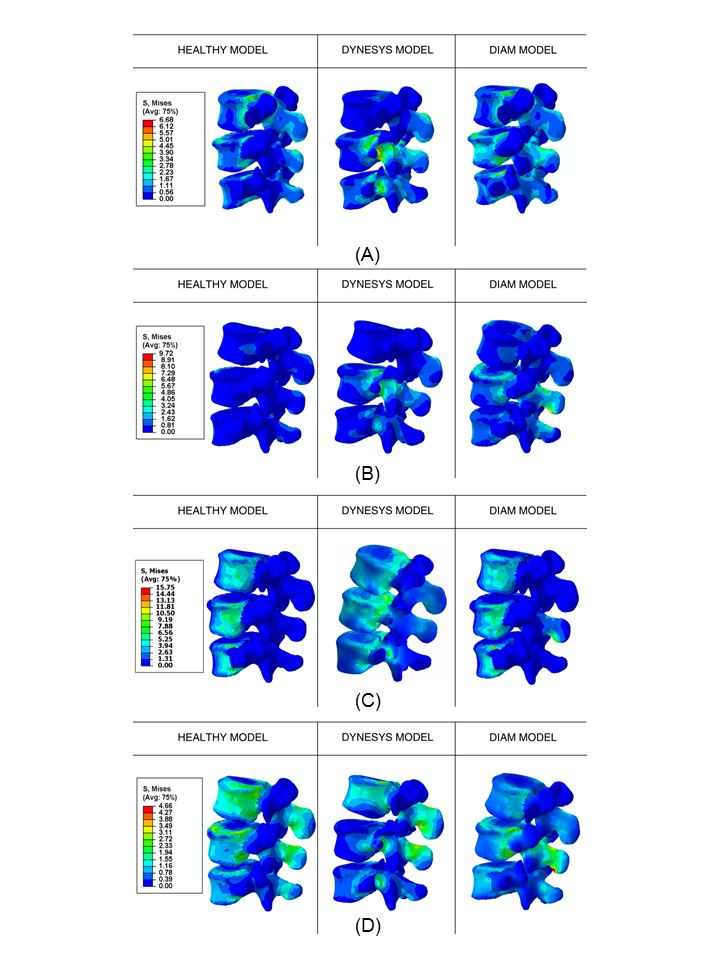

Supplement: S6 Fig — A, B, C and D. Von Mises stress maps in vertebras L3, L4 and L5 for healthy, DYNESYS and DIAM models. (A) Flexion. (B) Extension. (C) Lateral bending. (D) Axial rotation. (TIF) [file pone.0188328.s006.tif]

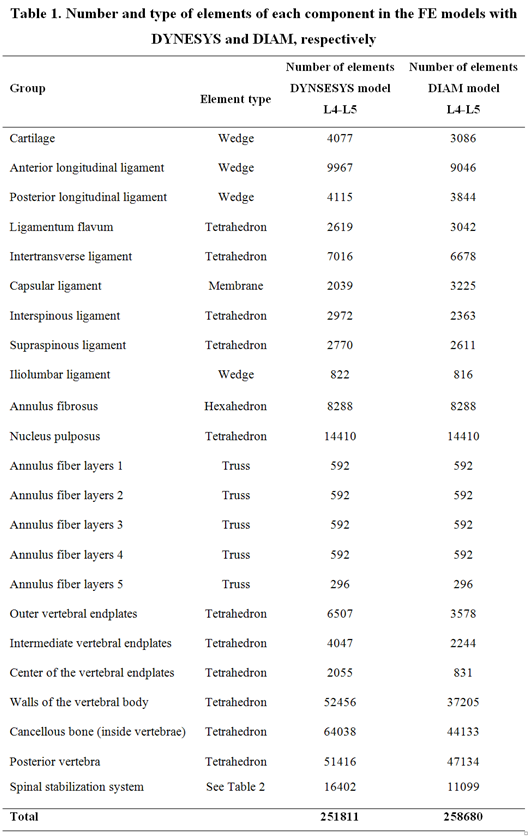

Supplement: S1 Table — (TIF) [file pone.0188328.s007.tif]

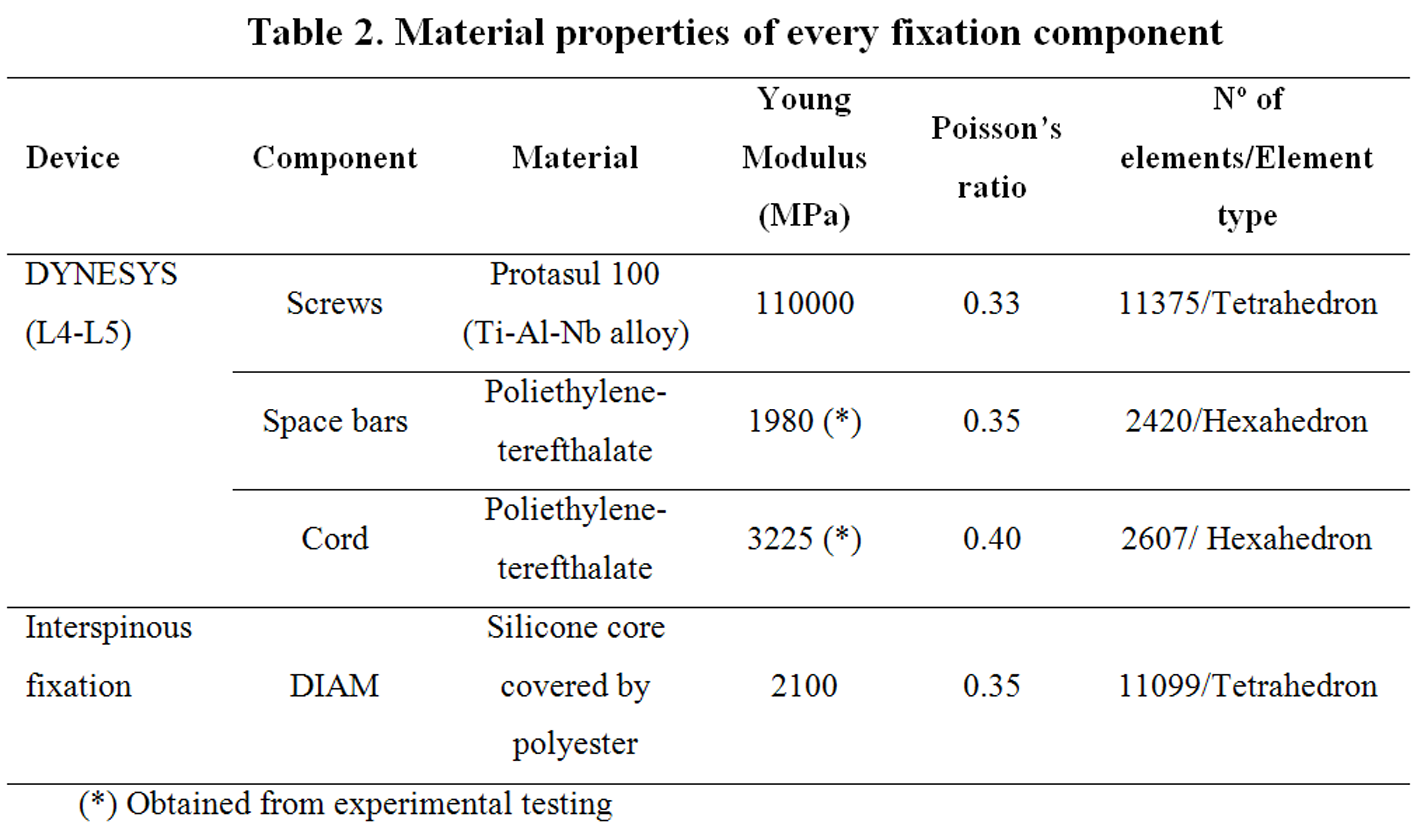

Supplement: S2 Table — (TIF) [file pone.0188328.s008.tif]

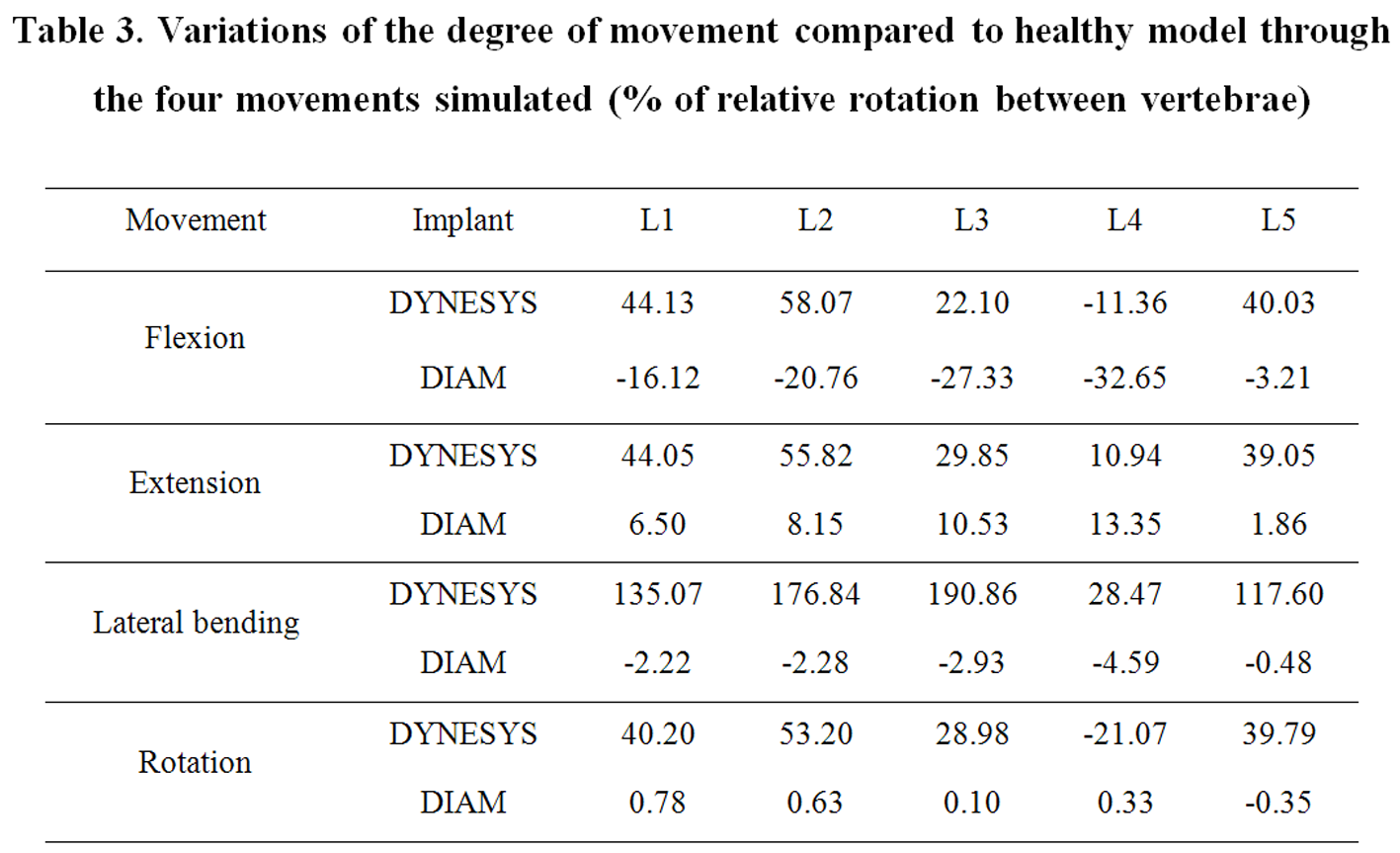

Supplement: S3 Table — (TIF) [file pone.0188328.s009.tif]
